# Cholera outbreak following a marriage ceremony in Medinya, Western Ghana

**DOI:** 10.11604/pamj.supp.2016.25.1.6167

**Published:** 2016-10-01

**Authors:** Helena Acquah, Keziah Malm, Joyce Der, Gideon Kye-Duodu, Ebenezer Kofi Mensah, Samuel Oko Sackey, Kofi Mensah Nyarko, Edwin Afari

**Affiliations:** 1 Ghana Field Epidemiology and Laboratory Training Programme, School of Public Health, University of Ghana, Ghana; 2 Veterinary Services Directorate, Accra, Ghana; 3 Diseases Control and Prevention Department, Ghana Health Services, Accra, Ghana; 4 University of Health and Allied Sciences, Ho, Volta Region, Ghana

**Keywords:** Cohort studies, cholera outbreak, serotype-ogawa, Ghana

## Abstract

**Introduction:**

Cholera is a diarrhoea disease caused by the bacterium *e*. On 13th June 2011, there was a reported outbreak of acute watery diarrhoea at Medinya among people who eat at a mass traditional wedding ceremony in the Western Region of Ghana. We investigated to characterize the outbreak, and implement control and preventive measures.

**Methods:**

We conducted a retrospective cohort study. We interviewed health workers, reviewed medical records, conducted environmental assessment and obtained water and stool samples for laboratory investigation. A suspected cholera-case defined as a person with acute watery diarrhoea, with or without vomiting, who ate food prepared at the mass traditional wedding in Medinya on 10th June 2011. We performed univariate and bivariate analysis.

**Results:**

Of the 17 case-patients, 9 (52.9%) were males. The overall attack rate was 11.18% and case fatality rate was 5.9%. The most affected age group was 6-10 years (23.53%) with median age of 20 and ranged 6 to 38 years. Time of onset of symptoms was 2.00am and peaked at 10.am on 13th June. Compared to other food served, fufu with groundnut soup was more likely to have been contaminated (RR=7.3, 95%CI: 1.8-29.3). We isolated *e* serotype ogawa from stool samples. We observed open defaecation and poor personal hygiene.

**Conclusion:**

*e* serotype ogawa caused a high case-fatality outbreak in Medinya. Contaminated fufu and groundnut soup were the sources. Hand washing with soap was initiated and a make shift latrine constructed following our health education and recommendations.

## Introduction

Cholera is an acute diarrhoeal infection transmitted through ingestion of food or water contaminated with the bacterium *e*. Two serogroups of *V. cholerae*, O1 and O139 cause outbreaks [1–3]. Cholera is an extremely virulent disease and affects both children and adults and can kill within hours. The short incubation period of two hours to five days, enhances the potentially explosive pattern of outbreaks [2]. For 75% of people infected with *V. cholerae*, no symptom develops, although the bacteria can be present in their faeces for 7-14 days after infection. The bacteria are shed back into the environment which potentially infects other people. Among people who develop symptoms, 80% have mild or moderate symptoms, while around 20% develop severe dehydration which can lead to death if untreated [1]. It is estimated that cholera affects 3-5 million people worldwide, and causes 100,000 to 130,000 deaths a year as of 2010 [1, 2]. In 2011, fifty eight countries notified World Health Organization (WHO), with a cumulative total of 589854 cases and 7816 deaths from all regions of the world. From the African continent, 188678 cases and 4183 deaths were reported, of which Ghana reported 10628 cases and 105 deaths [4]. From September 2010 to 15th June, 2011, Ghana reported outbreaks of cholera in 6 regions. The Western Region of Ghana had then not reported any case of cholera for the past 5 years [5–7]. Community marriage rites are practiced in parts of the Western Region. To cut down cost, young girls are included to the older ladies of the same family for the marriage rites. Traditional marriage ceremony of seven brides from same family took place on 10th June 2011. Visitors from 4 neighbouring villages attended the ceremony. On the 13th of June 2011, a number of people reported with acute profuse watery diarrhea at the sub-district hospital in Dixcove, Western Region of Ghana. They were diagnosed of having cholera and an outbreak was declared. An investigation and response team of the Ghana Field Epidemiology and Laboratory Training Programme was tasked to establish the extent of the outbreak, identify the source of infection and to support with implementation of control and preventive measures. This paper reports the findings of this investigation.

## Methods

### Study Design

A retrospective cohort study was conducted among persons, who ate food prepared at the mass traditional wedding in Medinya on 10th June, 2011. The investigation was carried out from 25th June to 1st July 2011.

### Study Site

**Figure 1:**
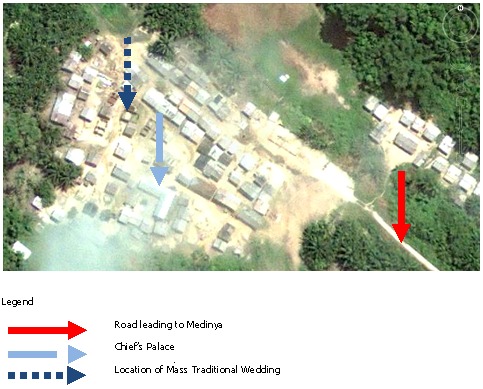
Overhead view of Medinya (courtesy, Google Earth)

Medinya is a community in the Ahanta West District of the Western Region of Ghana and has a population of 152 people within 68 households and enclosed by forest belt. The affected community is 8km from the nearest hospital in Dixcove sub-district. The only road that leads to this community is dilapidated, making it hard to reach area. The main economic activity is farming and sources of drinking water are two wells and a stream. The only toilet facility in the community had collapsed after a heavy rainfall. Figure 1 is a Google map of Medinya and its households.

### Data collection

We interviewed the Regional and Dixcove Sub district Disease Control Officers, Hospital Director of the main public hospital in Dixcove, the chief, local authority members and community members of Medinya in the Dixcove sub- district using an interviewer guide. In addition we reviewed hospital medical records to ascertain the number of diarrheal cases managed at the hospital for the preceding year and the period under study. We further obtained line lists from the health facility and the disease control office to validate the information. We defined a suspected cholera-case as a person with acute watery diarrhea, with or without vomiting, who ate food prepared at the mass traditional marriage ceremony in Medinya on 10th June 2011. A confirmed case was any person with a compatible clinical presentation, who ate food prepared at the mass traditional marriage ceremony in Medinya on 10th June 2011 with *e* confirmed by culture. Based on the working case definition, active case search was conducted to identify cases that might not have sought care from the hospital. Diagnostic records of cholera at the Zonal Public Health Laboratory were reviewed. We assessed the environment on sanitation and hygienic practices of community members by observation. We collected fresh water specimens from two wells (well 1 and well 2) and a stream and stool samples stored at the Zonal Public Health Laboratory to the National Public Health Reference Laboratory (NPHRL) for confirmation. Selected community members were interviewed using semi-structured questionnaires to obtain information on demographic characteristics, food handling practices, food that was eaten and water that was drunk during the ceremony. Other variables collected were, time of meal, types of symptoms, date and time of onset of symptoms and results of laboratory testing. Data collected was entered in Microsoft Excel and analyzed in Epi Info version 3.5.1. Descriptive and inferential analysis was carried out based on the initial information gathered. We performed univariate and bivariate analysis of outbreak data. Relative risk was used as measure of association at a significance level of 5%.

### Ethical statement

Prior permission was sought from the Regional Health Directorate before the study. Consent was obtained from Chief of Medinya and community members before interviews were conducted and participation was voluntary.

## Results

**Figure 2:**
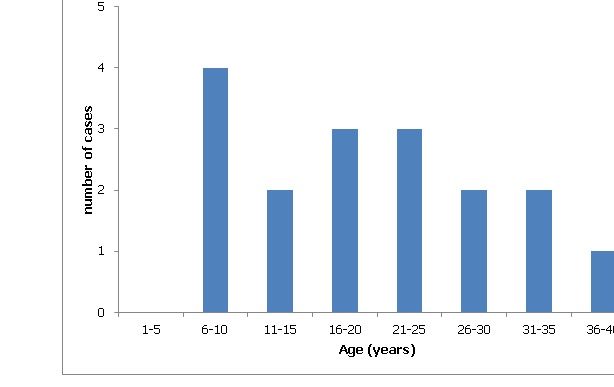
Cholera case patients by age, Medinya, Western Region, 13-19 June, 2011

The hospital and disease control officers presented a line list of 12 case-patients. Through the active case search additional five case-patients were identified. Of the 61 members interviewed, 17 (27.8%) case-patients were identified with 9 (53 %) being males. The affected ages ranged from 6-38 years with median age of 20. The most affected age group was 6-10 years (23.53%) and the least affected age group was 36 - 40 years (5.9%). Figure 2 shows the age distribution of cases. About 100 people feasted on food prepared and food was served mostly by visitors from 10.30am to 6.00pm. Source of water used was from 2 wells in the community. The overall attack rate was 27.8% (17/61) and case fatality rate was 5.9% (1/17). Most of the affected case-patients (76.5%) were from Medinya. Four visitors from adjourning communities, were identified as case-patients constituting 23.5% of the total number of cases. All these case-patients attended and ate foods prepared at the wedding ceremony in Medinya.

**Figure 3:**
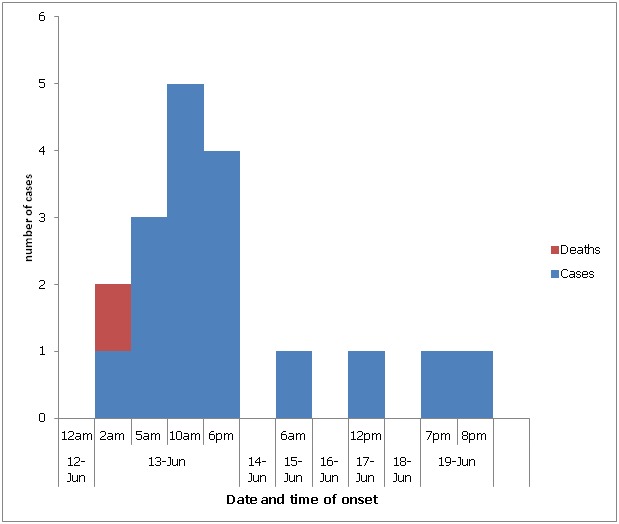
Cholera case patients by time of onset of symptoms, Medinya, Western Region, 13-19 June, 2011

Epidemic curve: the index case was a six year old bride; she developed diarrhoea at 2.00am on 13th, June, 2011, sought care at the sub-district hospital at 9.45 am the same day but died an hour later at the hospital. Time of onset of outbreak was at 2:00am and peaked by 10:00am same day. The number of cases declined until the 19th of June. There were no cases on the 14th, 16th, and 18th, of June. Figure 3 presents the epidemic curve.

Laboratory Confirmations: the well water was macroscopically dirty upon examination. From the laboratory investigation, six of seven stool samples from case patients tested positive for *e* Ogawa. Additional two stool samples from asymptomatic contacts were also positive. All water samples taken from the two wells and stream tested positive for *E. coli* but negative for *V. cholerae* Ogawa.

Environmental Findings: there was open defaecation in the community with refuse dumps close to houses. Washing hands with soap before eating and after defecation was not commonly practiced.

Analytical Epidemiology: the initial information gathered led to the hypothesis that, the cholera outbreak was likely to have occurred due to the contamination of a batch of food (fufu) that was served with bare hands by a “potential” carrier or by contaminated water. Of the 33 people who ate fufu with groundnut soup, 15 got ill: a food specific attack rate of 45.5%. Eating fufu with groundnut soup was significantly associated with falling ill at 95% confidence level (RR 7.3, 95%, CI: 1.8-29.3). There was no significant association between eating fufu with palm nut soup (RR 0.937, 95%, CI: 0.2-5.5), or eating rice (RR 0.00, 95%, CI: undefined) or drinking water from both well1 (RR 1.33, 95%, CI: 0.2-8.1) and well 2 (RR 0.38, 95%, CI: 0.1-0.9) respectively. Table 1 presents Food Specific Attack Rates at Medinya on 10th June, 2011.

**Table 1:** Food /water ingested at Traditional Wedding, Medinya, 10^th^ June, 2011

Item Served	No. of Persons who ATE specified food				No. of Persons who did NOT eat				
	Ill	Not Ill	Total	Attack Rate (%)	Ill	Not Ill	Total	Attack Rate (%)	Attack Rate Ratio
Fufu with Groundnut soup	15	18	33	45.5	2	30	32	6.3	7.3
Fufu	16	25	41	39.5	1	23	24	4.2	9.4
Groundnut Soup	15	27	42	35.7	2	21	23	8.7	4.1
Rice	0	2	2	0	17	46	63	26.9	0
Palm nut Soup	2	15	17	11.8	15	33	48	31.3	0.38
Well 1	16	44	60	25.0	1	4	5	20	1.25
Well 2	14	46	60	23.3	3	2	5	60.0	0.38
Rain water	1	4	5	20.0	16	44	60	26.7	0.75
Yam	0	3	3	0	17	45	62	27.4	0

## Discussion

The study revealed that, the cholera outbreak affected the young adults in the community and more especially among children probably due to their vulnerability. This finding is consistent with other reports [1, 8, 9]. Traditionally, children and men are served meals first in Medinya. This could have explained why more children and males were affected in this outbreak as most of the case patients ate the first batch of food prepared. The index case died shortly after reaching the hospital and this is likely to be due to the long period between onset of symptoms and arrival at the hospital as she was taken on foot to the hospital. The only road to the community is dilapidated and drivers of commercial vehicles are unwilling to take the risk to help transport patients. This study is consistent with a study in the Amazon where high case fatality of village cholera outbreaks was blamed on poor access to health facilities [10]. This was an outbreak of Cholera which remains a major public health problem in many parts of Ghana and now includes Western Region. Cholera is now thought to be endemic in parts of the country [5, 6, 11, 12]. Although this outbreak occurred after feasting at a traditional marriage ceremony, and the region had no cholera reported for more than five years, cholera was however endemic in other parts of the country and someone could have imported it to the community during the marriage ceremony which infected the food (fufu) during its preparation. Cholera continues to be an important public health problem among many poorer communities, though the detailed understanding of the bacteriology, epidemiology, and public health aspects are known for centuries [13]. The outbreak occurred in a very poor community, totally isolated from town with no social amenities. Asymptomatic people infected with *V. cholerae*, can be sources of cholera outbreaks in communities [1, 3]. The probable source of infection could also be from food which could have been contaminated by a “carrier” who served food during the marriage ceremony. Poor sanitation and poor personal hygiene has been identified as significant risk factors for cholera outbreaks [3, 11, 8]. Our findings is consistent with other reported outbreaks in other regions of Ghana and parts of Africa as washing hands with soap before eating and after defaecation was not commonly practiced by community members. To ensure timely health care, we recommended a Community-based Health Planning and Services (CHPS) compound which is a basic health post to be set up in the community to improve access to health care. To help prevent any further outbreaks, measures carried out in this study were in accordance with WHO recommendations [1]. We strengthened health education on sanitation and safe food handling practices through a house-to-house approach in Medinya. Collaboration with the Municipal Assembly facilitated the supply of Aqua tabs to chlorinate the wells.

**Limitations:** some of the food handlers were visitors from neighbouring communities and could not be located for interview and examination for any possible infections. The food handlers identified in Medinya had been given prophylactic treatment by the regional Health Team before our investigation was conducted. There were no food samples for laboratory analysis because all foods prepared at the ceremony had been consumed.

## Conclusion

* serotype Ogawa* caused a high fatality, point source post-traditional marriage ceremony outbreak in Medinya which affected mostly children aged 6 to 10 years. Fufu and groundnut soup was the source of the outbreak. Hand washing with soap was initiated and relocation of refuse dumps farther away from residential quarters and the construction of a make shift latrine by community members were facilitated.
